# Editorial: Interactions at the viral-host nexus in animals: from omics insights to immune modulation

**DOI:** 10.3389/fcimb.2025.1667802

**Published:** 2025-08-15

**Authors:** Shuliang Qu, Muhammad Munir, Jing Qian, Jia He, Jianzhong Wang, Xiaohong Xu

**Affiliations:** ^1^ State Key Laboratory for Diagnosis and Treatment of Severe Zoonotic Infectious Diseases, Institute of Zoonosis, College of Veterinary Medicine, Jilin University, Changchun, China; ^2^ Key Laboratory for Zoonosis Research of the Ministry of Education, Institute of Zoonosis, College of Veterinary Medicine, Jilin University, Changchun, China; ^3^ Faculty of Health and Medicine (FHM), Lancaster University, Lancaster, United Kingdom; ^4^ Institute of Veterinary Medicine, Jiangsu Academy of Agricultural Sciences, Nanjing, China; ^5^ Department Medicine and Dentistry, University of Alberta Department of Biological Sciences, Edmonton, AB, Canada; ^6^ Institute of Zoonosis, College of Veterinary Medicine, Jilin Agricultural University, Changchun, China

**Keywords:** muti-omics, algorithm innovation, emerging field, transcriptomic, proteomics

## Introduction

Multi-omics integration has emerged as a powerful approach for gaining insights into complex biological system. By combining data from genomics, transcriptomics, proteomics, metabolomics, or other omics layers. This strategy enables a richer understanding of host-pathogen interactions, disease mechanisms, and therapeutic targets. Current advancements include three key areas: applications of multi-omics integration, innovations in algorithmic approaches, and emerging fields where these techniques are being applied.

## Multi-omics integration applications

Multi-omics integration enhances the ability to characterize and manage pathogens by providing comprehensive data-driven insights into disease pathogenesis, biomarker discovery, and therapeutic interventions. In terms of disease diagnosis, multi-omics data—spanning genomic, transcriptomic, proteomic, and metabolomic layers—has significantly improved early detection and cancer subtype classification (Milner and Lennerz, 2024) (Zhao et al., 2024). Pathogen genomics plays a crucial role in monitoring and forecasting outbreaks, integrating methods allows experts to track the spread of pathogens and identify emerging threats, thereby supporting evolutionary and epidemiological studies (Knijn et al., 2023; Wu et al., 2024). Multi-omics data also reveals potential mechanisms underlying disease pathogenesis. For example, joint analysis of multi-omics data in chronic liver disease (CLD) or psychiatric conditions linked to infectious pathogens highlights novel aspects for drug development (Lin et al., 2022; Okobi et al., 2024). Zheng et al. integrated virome data of bronchoalveolar lavage fluid (BALF) and peripheral blood samples to provide novel insights into pulmonary microbial ecology (Zheng et al.). Shaji et al. *have* combined the data of kinase-substrate phosphomotif pattern analysis on the SARS-CoV-2 proteome with literature review and in-silico protein-protein docking approach, presents a framework for predicting SARS-CoV-2 phosphosites, suggesting MAP2K1 to be a key host kinase (Shaji et al.). Therefore, multi-omics integration provides a multidimensional view of diseases, significantly improving diagnostics, surveillance, and treatment strategies in pathogen-related contexts.

## Algorithm innovations in multi-omics integration

Algorithmic innovations are critical for overcoming challenges in multi-omics data analysis, such as handling high-dimensional datasets, integrating diverse omics layers, and improving predictive accuracy. Recent advances leverage deep learning, machine learning, and novel computational frameworks to enhance integration. Deep learning has revolutionized multi-omics integration by enabling feature learning and flexible data fusion strategies. Methods like MoAGL-SA introduced adaptive integration techniques for cancer subtype classification, addressing key issues such as embedding sample structure information into the feature space (Cheng et al., 2024). Supervised deep generalized canonical correlation analysis (SDGCCA) is another innovation that models nonlinear correlations between omics manifolds, improving phenotype classification and revealing relevant biological mechanisms (Moon et al., 2022). These approaches categorize architectures—such as neural networks—that handle complex, non-linear relationships in multi-omics data, facilitating more robust predictions for disease subtypes and biomarker identification (Ballard et al., 2024; ElKarami et al., 2022). Innovations in single-cell multi-omics integration allow for joint analysis at the resolution of individual cells, providing insights into cellular heterogeneity and complex traits like therapeutic resistance and immune evasion. Techniques focus on integrating omics data at the single-cell level, making them accessible for non-experts and enhancing understanding of cell states and gene functions (Hu et al., 2024; Xiao et al., 2024). Additionally, machine learning-based natural language processing (NLP) algorithms are employed to automate the extraction of pathogen-specific information from literature, such as detecting papers on experimental pathogen studies. Tools such as READBiomed-Pathogens train models to identify and characterize mentions of pathogens in scientific texts, filling gaps in manually annotated corpora and accelerating knowledge discovery (Jimeno Yepes and Verspoor, 2023).These innovations address challenges like data dimensionality reduction, integration of sparse datasets, and the incorporation of machine learning for refinement, thereby enabling more intuitive and informative interpretations in pathogen research (Ngai et al., 2025).

## Emerging fields of application

Driven by technological advancements, omics are expanding into novel aspects. For instance, the integration of multi-omics with electronic health records (EHR) and clinical imaging represents a frontier in precision health, allowing for disentangling host-microbe and host-environmental interactions (Babu and Snyder, 2023; Lin et al., 2022). Wang et al. integrated proteomics and phosphoproteomics of freshwater mollusks to provide novel insights as potential food source (Wang et al.). Additionally, Li et al. innovatively utilized humanized major histocompatibility complex (hMHC) murine models to reflect immune responses in SARS-CoV-2 infection, showing elevated immune response, especially IFN-γ signaling pathways and neutrophil mobilization dynamics that closely parallel human post-vaccination responses, confirmed that the hMHC murine model can serve as an effective platform for vaccine research (Li et al.). Chen et al. applied transcriptomic analysis to aquatilia, explained Chinese mitten crab immunity against bacterial infection, showing the differences between disease-resistant and susceptible species (Chen et al.). Research on novel or understudied pathogens emphasizes the need for multidisciplinary approaches, such as collaborations across animal, plant, and human disease contexts to understand cross-sectional involvement.

## Conclusion

Multi-omics integration has become a cornerstone of modern pathogen research, offering unprecedented capabilities in disease understanding, algorithmic processing, and cross-disciplinary applications. Through innovations in deep learning and machine learning-based algorithms, researchers can now better integrate complex datasets for improved disease prediction and pathogen-host interaction. Emerging fields, such as radiopathomics and spatial multi-omics, demonstrate the versatility of this approach, extending beyond human health into agriculture and environmental science. However, challenges still remain, including data integration hurdles and the need for high-quality annotations. Future directions emphasize collaborative efforts to refine multi-omics strategies and accelerate their implementation. Overall, these advancements highlight the transformative potential of multi-omics integration in unlocking new frontiers in pathogen biology.
